# Comparative study on the quality control effectiveness of AI-PBRTQC and traditional PBRTQC model in identifying quality risks

**DOI:** 10.11613/BM.2024.020707

**Published:** 2024-06-15

**Authors:** Xucai Dong, Xi Meng, Bin Li, Dongmei Wen, Xianfei Zeng

**Affiliations:** 1 Xi’an Area Medical Laboratory Center, Xi’an, Shaanxi, P.R. China; 3 School of Medicine, Northwest University, Xi’an, Shaanxi, P.R. China; 2 Shanghai Senyu Medical Technology Co., LTD, Shanghai, P.R. China

**Keywords:** patient-based real-time quality control, exponentially weighted moving average, quality risk

## Abstract

**Introduction:**

We compared the quality control efficiency of artificial intelligence-patient-based real-time quality control (AI-PBRTQC) and traditional PBRTQC in laboratories to create favorable conditions for the broader application of PBRTQC in clinical laboratories.

**Materials and methods:**

In the present study, the data of patients with total thyroxine (TT4), anti-Müllerian hormone (AMH), alanine aminotransferase (ALT), total cholesterol (TC), urea, and albumin (ALB) over five months were categorized into two groups: AI-PBRTQC group and traditional PBRTQC group. The Box-Cox transformation method estimated truncation ranges in the conventional PBRTQC group. In contrast, in the AI-PBRTQC group, the PBRTQC software platform intelligently selected the truncation ranges. We developed various validation models by incorporating different weighting factors, denoted as λ. Error detection, false positive rate, false negative rate, average number of the patient sample until error detection, and area under the curve were employed to evaluate the optimal PBRTQC model in this study. This study provides evidence of the effectiveness of AI-PBRTQC in identifying quality risks by analyzing quality risk cases.

**Results:**

The optimal parameter setting scheme for PBRTQC is TT4 (78-186), λ = 0.03; AMH (0.02-2.96), λ = 0.02; ALT (10-25), λ = 0.02; TC (2.84-5.87), λ = 0.02; urea (3.5-6.6), λ = 0.02; ALB (43-52), λ = 0.05.

**Conclusions:**

The AI-PBRTQC group was more efficient in identifying quality risks than the conventional PBRTQC. AI-PBRTQC can also effectively identify quality risks in a small number of samples. AI-PBRTQC can be used to determine quality risks in both biochemistry and immunology analytes. AI-PBRTQC identifies quality risks such as reagent calibration, onboard time, and brand changes.

## Graphical abstract

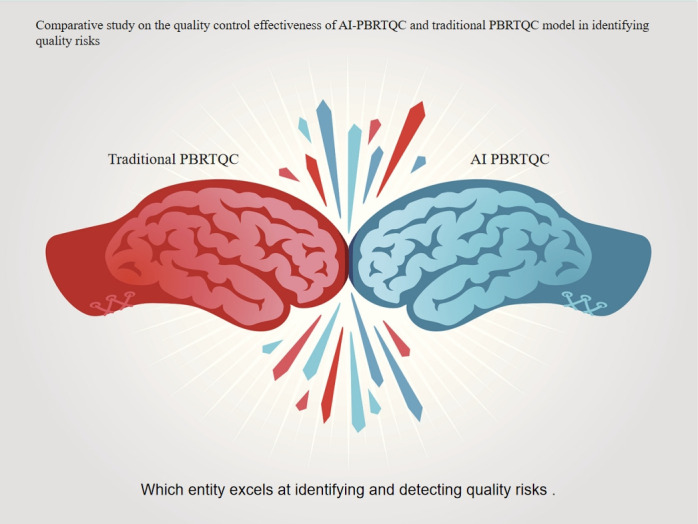

## Highlights

• Establish optimal patient-based real-time quality control models for different analytes based on patient-based real-time quality control real-time intelligent monitoring platform

• Clinical application effect of artificial intelligence patient-based real-time quality control real-time intelligent monitoring platform in identifying real-world quality risks

## Introduction

In 1965, Hoffman *et al.* employed the “average of normals” methodology to facilitate the daily quality monitoring of laboratories, thereby initiating the practice of patient-based real-time quality control (PBRTQC) in laboratory medicine (*1*). Subsequently, the Bull method was widely used for quality control of blood cell analysis (*2*). Patient-based real-time quality control is an innovative laboratory quality control method whose primary purpose is to assist internal quality control (IQC) in ensuring the accuracy and reliability of patient test results (*3*). Patient-based real-time quality control offers several notable advantages compared to IQC. These advantages encompass cost reduction, elimination of matrix effects, and continuous monitoring in real-time (*4*-*9*). In recent years, PBRTQC has witnessed a significant rise in its application within clinical laboratory settings. The adoption of this approach has gained significant traction owing to its inherent benefits, such as real-time process monitoring and the potential for cost reduction. The International Federation of Clinical Chemistry (IFCC) has proposed six PBRTQC algorithms as recommended guidelines. These algorithms encompass various statistical techniques such as the moving average (MA), moving median (MM), exponentially weighted moving average (EWMA), moving standard deviation (MovSD), and moving sum of outlier (MovSO) (*10**, **11*).

The achievement of a uniform standard to promote this complex PBRTQC is challenging. According to Badrick *et al.*, introducing PBRTQC necessitates a shift in the statistical process from empirical selection to future AI selection (*3*). These studies contribute to the expansion of potential applications for PBRTQC. Therefore, we need to compare the applicability of traditional PBRTQC and artificial intelligence (AI) PBRTQC in clinical laboratories.

In this study, we focused on: a) evaluating the ability of traditional PBRTQC models to identify quality control risks, b) evaluating the ability of the AI-PBRTQC model to identify quality risks and monitor its performance as a real-time quality control tool; c) comparing the AI-PBRTQC model with the traditional PBRTQC model regarding its ability to identify quality risks in the real world; 4) evaluating the application value of AI-PBRTQC and providing reference and practical experience for the application of AI-PBRTQC.

## Materials and methods

### Data source

From December 2021 to October 2022, the PBRTQC intelligent monitoring platform was installed and used on the local server of the laboratory to automatically collect patient data for total thyroxine (TT4), anti-Müllerian hormone (AMH), alanine aminotransferase (ALT), total cholesterol (TC), urea and albumin (ALB) in Xi’an Regional Medical Laboratory Center. The extracted data strictly complied with relevant laws and regulations and IFCC recommendations for PBRTQC and patient information identification was removed. The data did not involve the clinical privacy of patients and other ethical issues. The analytes selected for this experiment include chemiluminescence, rate, and endpoint methods. A wide range of biological variability was selected for this experiment. Patient specimens included pathological and health examination specimens, and the age of patients ranged from 2 to 98 years. The verified data ranged from a few hundred to tens of thousands. The selection of analytes and data described above ensured the study’s reliability, the sample’s diversity, and the results’ broad applicability. The results of the first six months served as a practice data set for parameter setting and procedure establishment of the PBRTQC model, and the results of the last five months served as a validation and evaluation data set for this study. All IQC results and test times were exported from the laboratory information system. Quality risk cases data for the study period were exported directly from each analyzer.

### Patient-based real-time quality control model

The data for the traditional PBRTQC model needed first to be subjected to the Box-Cox method of estimating the truncation range. The Box-Cox transform was used to estimate the truncation range of the data by the standard maximum likelihood method implemented in the optimal normalization of the R package (MASS) (*12*, *13*). Since most of the patient data were skewed, they needed to be transformed for Box-Cox data, and truncated ranges selected, but such truncated ranges may result in the loss of valuable data.

Artificial intelligence PBRTQC determined the appropriate truncation range based on the values of the project’s biological coefficient of variation and raw data. Monitoring used proprietary intelligent quality control rules that relyed on AI image recognition. If the data showed signs of deviating from the acceptable range, an alarm was activated or went out of control. Utilizing an advanced AI monitoring platform is essential for AI-PBRTQC. Artificial intelligence image recognition is our approach to image recognition and deep learning by continuously building models and selecting models with sensitivity and specificity greater than 96% for monitoring.

### Methods

Two Beckman DXI-800 and one Beckman AU5800 analyzer (Beckman Coulter, Brea, USA) are implemented in the Xi’an Area Medical Laboratory Center. Furthermore, we adopted the PBRTQC platform from Shanghai Senxu Medical Technology Co. and the LTD intelligent monitoring platform. Patient results were collected by the laboratory information system of Shanghai XingHE Software Co., LTD. The outliers of the patient data from the traditional PBRTQC group were truncated by Minitab 20.0 (State College, Pennsylvania). In the Box-Cox transformation using the R package (MASS), data close to the normal distribution were selected as the truncation range. The Box-Cox transform was performed using the R package (best normalized), and data close to normal distribution was selected as the truncated range. Error detection (Ped), false positive rate (FPR), false negative rate (FNR), and average number of the patient sample until error detection (ANPed) data were analyzed using WPS Excel 2023 (Xi’an, Shaanxi Province, China). Receiver operating characteristic (ROC) curves were plotted using IBM SPSS Statistics for Windows version 20.0 (IBM Corp.). Biological variation data were extracted from the European Federation of Clinical Chemistry and Laboratory Medicine (EFLM) biological variation database (*14*).

**Figure 1:**
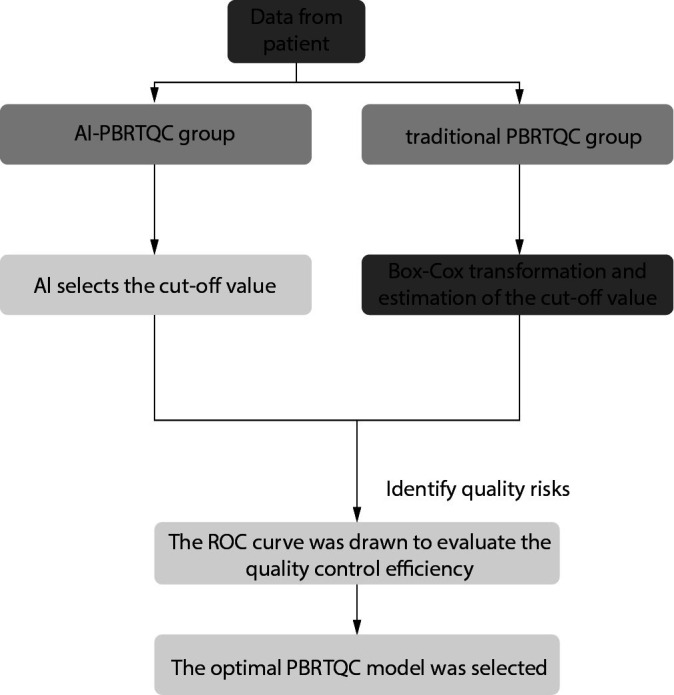
Data processing of the two PBRTQC groups. PBRTQC - patient-based real-time quality control. AI - artificial intelligence. ROC - receiver operating characteristic.

In this experiment, different truncation ranges for traditional PBRTQC and AI-PBRTQC were calculated, as shown in Figure 1. Then parametric models with different truncation ranges and weighting coefficients were built on the PBRTQC platform to monitor the patient data for the same period. The AI-PBRTQC and traditional PBRTQC groups identified real-world quality control risky cases and evaluated the optimal PBRTQC scenarios by Ped, FPR, FNR, ANPed, and area under the curve (AUC), FNR, ANPed, and AUC to assess the optimal PBRTQC protocol.

### Quality management system

Calibration, IQC, and instrument maintenance were performed by the standardized management requirements of the ISO 15189 quality management system.

### Patient-based real-time quality control algorithm

The intelligent monitoring platform AI-PBRTQC received the test results of laboratory patients from the LIS. Patient-based real-time quality control operating program algorithm: EWMA algorithm using AI-PBRTQC intelligent monitoring platform. The calculation model of the EWMA control chart was as follows: 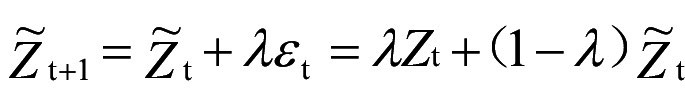


(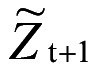 represented the estimate of point t+1, 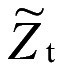 represented the estimate of point t, represented the actual measurement of point t, λ is the weighted coefficient, 0 < λ < 1).

### Quality control rules

The IQC utilized Westgard rules 1-2S, 1-3S, 2-2S, R-4S, and 10-X. The PBRTQC software incorporated intelligent quality control rules founded on artificial intelligence image recognition from the AI-PBRTQC intelligent monitoring platform and the conventional Westgard rules (*15*, *16*).

### Calibration events

The timing of calibration events was not based on the results of IQC or PBRTQC but rather determined solely by the laboratorian based on the current status of the analyzers. A positive calibration event is achieved when the IQC warning or loss of control occured or when the trend of the EWMA curve was reversed after calibration events. Otherwise, the calibration was considered a negative event.

### Quality risk cases

Quality risks encompassed a range of potential hazards arising from reagent opening times, variations between reagent bottles, discrepancies in batch numbers, expiration of weekly maintenance cleaning solutions, and other alterations. Data collected from the PBRTQC software was analyzed using quality risk cases as a gold standard benchmark. If the EWMA curve exhibited a warning or went out of control during a quality risk incident, then the warning or out of control was classified as a true positive. The time between the occurrence of a quality risk event and its correction was called the quality risk period. If there were no warnings on the PBRTQC platform, this risk event was considered a false negative. Whenever the EWMA curve showed a warning or went out of control despite no quality risk event present, then the warning or out of control was a false positive. Conversely, when there was no warning, it was correctly identified as a true negative.

### Corrective measures

When a quality risk arises within a laboratory, it is imperative for the laboratory to promptly identify the underlying cause and implement appropriate measures to rectify the situation. Measures encompassed various actions such as calibration, reagent replacement, equipment troubleshooting, and other related procedures.

### The optimal PBRTQC model

The optimal PBRTQC model was selected by Ped, FPR, FNR, ANPed, and AUC for the two sets of data from the intelligent monitoring platform. Ped is defined as the probability that a quality control rule and its combination can effectively detect an analytical error when it occurs in routine analysis, equivalent to the sensitivity of a clinical diagnostic test. The ideal Ped for a quality control method should be 1.00; that is, 100% of the analytical batch can detect errors. In the actual operation of clinical testing quality control, Ped between 90-99% is generally considered acceptable. False positive rate was defined as the proportion of all true negative samples incorrectly judged as positive by the model. False negative rate was defined as the proportion of all true positive samples which were incorrectly determined as negative by the model. The number of patient sample results required before error detection was statistically derived from the software’s backend data was defined as ANPed. The lower the ANPed, the more sensitive the PBRTQC model to identify errors.

Optimal PBRTQC models for different analytes based on the PBRTQC real-time intelligent monitoring platform were established. The choice of PBRTQC parameters should meet both the high error detection rate and the low false rejection rate. Generally, the general clinical laboratory requires the error detection rate to be > 90%, while the false rejection rate is < 5%. This study used the ROC curve to evaluate the performance of the artificial intelligence PBRTQC model and traditional PBRTQC model procedures. Graph used FPR as abscissa and true positive rate (TPR) as ordinate. Different models generated different points at different thresholds, which were connected to form ROC curves. The AUC was used as the leading indicator to evaluate the performance of different methods, and the best performance in quality control was judged by determining and comparing the AUC values of different methods. The evaluation criteria were as follows: AUC > 0.9 indicated excellent method performance and significant diagnostic efficiency in identifying quality risks (*17*-*20*). This study combined the AUC and the above indicators to select the optimal model. The AUC can observe the quality control efficiency of the model.

## Results

### Patient-based real-time quality control parameters

Six routine analytes were evaluated in the present study, encompassing diverse biological variants, distinct methodologies, and varying quantities of data. Supplementary Figure 1 shows the resultant graphs of the EWMA algorithm for the PBRTQC model with different weighting factors. Supplementary Figure 2 shows the ROC curve graphs for identifying quality risks with different weighting factors. The results of the analysis of the two sets of PBRTQC models with different weighting coefficients are shown in Table 1. According to Table 1, the optimal models for PBRTQC are all those that have been identified in bold and are all in the AI-PBRTQC group. The between-subject biological variation (CVg) and within-subject biological variation (CVi) for TT4 and ALB are relatively small, and the weighting coefficients of their optimal PBRTQC models for all other analytes are 0.05. The weighting coefficients for all other analytes are 0.02.

**Table 1:** Quality control parameters of the patient-based real-time quality control in the two groups

**Analyte**	**CVg**	**CVi**	**Truncation** **range**	**Number of PBRTQC platform alarms**	**Data volume**	**Weighting** **coefficient (λ)**	**False detected alarm data**	**Ped (%)**	**FPR (%)**	**FNR** **(%)**	**ANPed**	**AUC**
TT4 (nmol/L)*	11.8	6.4	**78-186**	336	1315	0.02	0	0	0	100	-	0.911
						0.03	0	80.95	0	19.05	8	0.913
						**0.05**	**26**	**92.26**	**2.6**	**7.74**	3	0.921
TT4 (nmol/L)^†^			82.15-200.19	334	1431	0.02	0	0	0	100	-	0.843
						0.03	0	0	0	100	-	0.839
						0.05	117	69.16	10.67	30.84	5	0.834
AMH (ng/mL)*	/	19.2	**0.02-2.96**	314	734	**0.02**	**0**	**96.82**	**0**	**3.18**	**2**	**0.887**
						0.03	199	66.56	47.38	33.44	35	0.837
						0.05	82	28.03	19.52	71.97	40	0.759
AMH (ng/mL)^†^			0.02-10.14	501	1125	0.02	97	41.52	15.54	58.48	68	0.418
						0.03	6	41.32	0.96	58.68	71	0.419
						0.05	221	39.52	35.42	60.48	80	0.436
ALT (U/L)*	29.3	10.1	**10-25**	1856	11343	**0.02**	**173**	**96.98**	**1.82**	**3.02**	**6**	**0.916**
						0.03	178	77.75	1.88	22.25	8	0.882
						0.05	954	72.14	10.06	27.86	0	0.834
ALT (U/L)^†^			6-29	2185	12284	0.02	1520	90.94	15.05	9.06	0	0.894
						0.03	3394	32.72	33.61	67.28	0	0.859
						0.05	3819	48.88	37.82	51.12	0	0.817
TC (mmol/L)*	16.7	5.3	**2.84-5.87**	2143	2697	**0.02**	**0**	**90.85**	**0**	**9.15**	**24**	**0.957**
						0.03	0	72.52	0	27.48	32	0.947
						0.05	50	59.4	9.03	40.60	44	0.948
TC (mmol/L)^†^			2.79-6.4	2345	2929	0.02	129	80.94	22.09	19.06	0	0.894
						0.03	179	76.67	30.65	23.33	0	0.859
						0.05	243	63.8	41.61	36.20	0	0.817
UREA (mmol/L)*	21.0	13.9	**3.5-6.6**	246	3083	0.02	**0**	**92.28**	**0**	**7.72**	**5**	**0.744**
						0.03	135	76.02	4.76	23.98	0	0.735
						0.05	350	71.54	12.34	28.46	0	0.719
UREA (mmol/L)^†^			2.3-7.68	210	3116	0.02	2258	61.9	77.7	38.10	0	0.322
						0.03	1019	13.81	35.07	86.19	0	0.370
						0.05	882	48.1	30.35	51.90	0	0.412
ALB (g/L)*	4.9	2.5	**43-52**	717	10071	0.02	0	0	0	100.00	-	0.723
						0.03	0	0	0	100.00	-	0.734
						**0.05**	**0**	**90.66**	**0**	**9.34**	**12**	**0.745**
ALB (g/L)^†^			41.9-53.1	717	11468	0.02	1454	0	13.5	100.00	-	0.715
						0.03	3197	69.18	0	30.82	0	0.726
						0.05	1872	43.51	17.41	56.49	0	0.727
*The artificial intelligence patient-based real-time quality control (AI-PBRTQC) group. ^†^Traditional patient-based real-time quality control (PBRTQC) group. CVg - between-subject biological variation. CVi - within-subject biological variation. λ - weighting coefficient of the exponentially weighted moving average (EWMA) method. Ped - number of alarms detected/number of PBRTQC platform alarms. FPR - false positive ratio (FP) / (FP + true negative (TN)). FNR - false negative ratio (FN) / (true positive (TP) + FN)). The optimal PBRTQC model is presented as bolded. TT4 - total thyroxine. AMH - anti-Müllerian hormone. ALT - alanine aminotransferase. TC - total cholesterol. ALB – albumin.												

**Figure 2:**
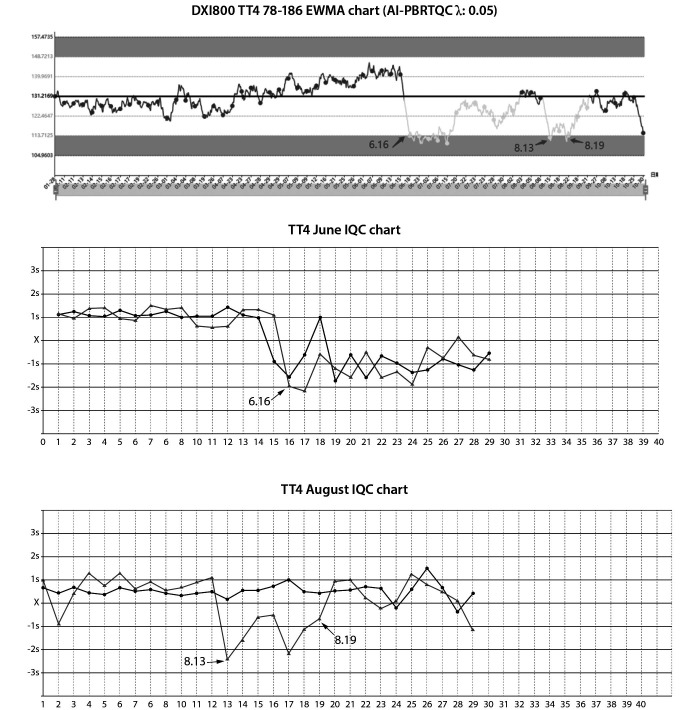
The PBRTQC and IQC chart of TT4. EWMA graphs in dark gray for no quality risk and light gray for detected quality risk. IQC graphs in black for low-level IQC and gray lines for high-level IQC. IQC – internal quality control. PBRTQC - patient-based real-time quality control. EWMA - exponentially weighted moving average. TT4 - total thyroxine.

**Figure 3:**
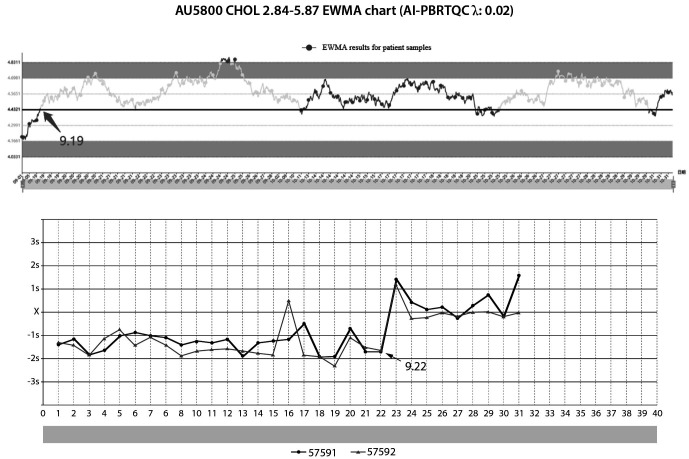
September IQC and PBRTQC EWMA chart for TC. EWMA graphs in dark gray for no quality risk and light gray for detected quality risk. IQC graphs in black for low-level IQC and gray lines for high-level IQC. IQC – internal quality control. PBRTQC - patient-based real-time quality control. EWMA - exponentially weighted moving average. TC – total cholesterol.

The quality risks are listed in Table 2, resulting in the findings presented in Table 1. This study PBRTQC identified nine quality risks, six of which occur quality risks due to calibration. As depicted in Figure 2, the validation of the TT4 case demonstrates that the positive calibration event significantly impacts the test results. Figure 2 shows the change in PBRTQC for TT4 due to calibration events versus IQC events. As shown in Figure 3, TC suggested a significant bias in IQC on September 22, while an alarm had occurred on the PBRTQC intelligent monitoring platform on September 19. This indicates that PBRTQC is more sensitive than IQC. After field verification, due to the reduced sample volume in the laboratory, this reagent was opened on September 22 and has been used for 23 days. After changing the new reagent, the PBRTQC platform returned to normal on October 31. Figure 3 shows the change in PBRTQC versus IQC due to the calibration event of the TC.

**Table 2:** The patient-based real-time quality control checklist for identifying quality risks

**Analyte**	**quality risk**			
**Cause**	**Date of occurrence**	**Date of closure**	**Corrective measure**
TT4 (nmol/L)	Periodic calibration	June 16	August 4	Calibration
TT4 (nmol/L)	Reagent replacement batch number	August 13	August 19	Calibration
AMH (ng/mL)	Reagent replacement batch number	June 6	July 28	Calibration
ALT (U/L)	Replace the new reagent brand	September 21	September 27	Use old brand reagents
ALT (U/L)	Replace the new reagent brand	October 9	October 31	Uncorrected, re-verify the data
TC (mmol/L)	Change calibrator batch number	August 3	August 25	Replace the calibrator and the sample back test
TC (mmol/L)	Reagent has a long bottle opening time	September 22	October 31	Replacement reagent
UREA (mmol/L)	Reagent replacement batch number	June 1	June 10	Calibration
ALB (g/L)	Reagent replacement batch number	June 27	June 30	Calibration
TT4 - total thyroxine. AMH - anti-Müllerian hormone. ALT - alanine aminotransferase. TC - total cholesterol. ALB – albumin.				

**Figure 4:**
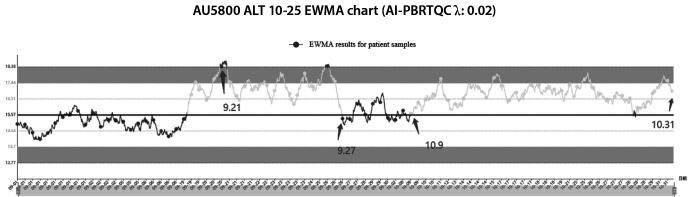
PBRTQC EWMA chart for ALT. EWMA graphs in dark gray for no quality risk and in light gray for detected quality risk. PBRTQC - patient-based real-time quality control. EWMA - exponentially weighted moving average. ALT - alanine aminotransferase.

As shown in Figure 4, the laboratory began contingency planning on September 21 to use a substitute brand of reagent because the logistics of the original brand of ALT reagent did not arrive in time. The laboratory continued to use the original brand of reagents after the supply was restored on September 27. On October 9, a departmental discussion led to the decision to use the alternative reagents as a long-term replacement for the original reagents. Figure 4 illustrates the change in PBRTQC from September 21 to October 31 for ALT on the quality risks that may result from changing reagents.

## Discussion

In this study, we aimed to compare the efficiency of AI-PBRTQC and conventional PBRTQC for quality control in laboratories and assessed its potential role in daily work. Artificial intelligence PBRTQC can be used as an effective tool for monitoring the quality control of laboratory tests.

According to our results, the optimal models for PBRTQC were all in the AI-PBRTQC group, which proves that the models built by the AI-PBRTQC are more suitable for these analytes. The reason may be that the algorithm of the AI-PBRTQC group can intelligently select the appropriate range of truncation based on the biological variability value of the analyte and the reference range data, which is more suitable for the nature of the patient data. We found that selections with considerable biological variation have a smaller weighting factor and use the weighting factor as a smoothing factor to reduce the effect of variation. However, of course, more cases are still needed to support this. The traditional PBRTQC model selects the truncation range by performing a Box-Cox transformation on the data. According to our results, using the truncation range of the traditional PBRTQC group produces many false positives, which is one of the main reasons why PBRTQC has not been widely used before. Rossum *et al.* also mentioned an alarm rate of 1% for PBRTQC, resulting in 30 unmanageable daily alarms (*9*).

The analytes selected in this study include biochemical and immunological analytes, which can effectively warn of quality risks. It proved that biochemical or immunological analytes can use the AI-PBRTQC software platform. Song *et al.* demonstrated that PBRTQC can monitor biochemical and immunoassay analytes *(**9**, **21**, **22**).*

The patient data for the analyte in this study ranged from 734 to 11,343. We proved that AI-PBRTQC is also effective in identifying quality risk in a small number of samples. Small sample size cases are less reported at this time.

Several studies have described methods to optimize PBRTQC models for implementing PBRTQC in routine clinical settings (*6*, *8*, *10*, *23*). Others have demonstrated the utility of PBRTQC for detecting temporary instrument failures in case reports, long-term assay stability issues, or interchangeability and lot-to-lot variability issues (*24*-*28*).

The validation of the TT4 case showed that the positive calibration event significantly affected the test results. However, after the calibration on 13 August 2022, we could only observe a relatively small change in the IQC. This insensitive change may be due to the limitations of the IQC, which only measures at the beginning of the analysis batch in the IQC and is related to the adjustment of the target value or SD of the IQC chart. This is why IQC used in the laboratory is not sensitive to calibration events. In contrast, PBRTQC is much less influenced by the laboratory than IQC and is more realistic. Our study is consistent with the findings of Song *et al.*, showing that PBRTQC shows greater sensitivity to bias in test results due to calibration events or analytical trends (*29*).

According to our results, if a low specimen volume caused the reagent to sit on the instrument for an extended period, the PBRTQC would raise the alarm, but the IQC just to one side. Reagents generally state an open box expiration date and a shelf life. The expiration date of chemical reagents varies significantly with the chemical nature. Generally speaking, the longer the shelf life of a chemically stable substance, the simpler the storage conditions. More factors, such as air, temperature, light, impurities, *etc.*, also affect reagents. Unstable reagents like disproportionation polymerization, decomposition, or precipitation may change after long-term storage. In the validity period of the liquid, if we find the reagents have delamination, turbidity, discoloration, mold, and other abnormalities, it should be a timely replacement of reagents.

We also demonstrated that PBRTQC can actively identify potential quality risks in reagent replacements. This case reminds us that reagent replacement requires performance validation and feedback from the clinical department.

Traditional PBRTQC has some advantages. It does not need a professional analytical platform, and even some laboratories use EXCEL for the construction of traditional PBRTQC model. Furthermore, traditional PBRTQC can achieve the quality of the whole process of monitoring. The disadvantages are the high rate of false positives, small specimen volume of the test items cannot be used, the model is more cumbersome to build. The advantages of AIPBRTQC are that it can realize the quality monitoring of the whole process; low false-positive rate, small specimen volume of test items can be used, items with sizeable biological variability can be used, and the model building is intelligent. The disadvantages are that the AI needs to be allowed to learn more cases to optimize the model in the early stage, and a professional monitoring platform is required.

The limitations of our investigation are as follows. The data obtained from PBRTQC extraction results is influenced by a multitude of factors to which patient specimens are routinely exposed to (*24*). These factors encompass a range of variables, such as atypical outcomes, samples obtained from diverse populations, a limited timeframe for data retrieval, varying clinical interventions, seasonal variations, specimen collection, transportation, and pre-processing, among other considerations. For example, the traditional PBRTQC had a truncated upper limit of 10.14. Although it was very high, it had a mean of 2.45, proving that most values were close to 2.45. Artificial intelligence PBRTQC has a much smaller amount of data than traditional PBRTQC, which may have something to do with excluding many results. During the analysis, we found that the data for AMH were heavily skewed to one side, which may be related to the fact that AI-PBRTQC discarded a large portion of the data.

There is currently a lack of consensus regarding the standardization of research on PBRTQC. This field encompasses a range of aspects, including simulation calculation methods, parameter optimization, evaluation indices, and validation methods. Hence, the absence of a comprehensive research framework presents challenges for researchers when it comes to comparing new methods with existing ones and facilitating comparisons among different researchers. When a researcher introduces a novel method, there is a dearth of a comprehensive research framework for conducting a comparative analysis with existing methods. Consequently, the credibility of the performance enhancement achieved by the new method cannot be assessed by other researchers. The scientific community has yet to achieve a comprehensive understanding of the performance enhancement associated with the new method (*25*). Difficulties in the application of PBRTQC arise from the complexity of simulating and optimizing parameters. These tasks require researchers with a strong background in computer science to complete them. Furthermore, the extension of PBRTQC to practical applications necessitates the support of inspection experts. Additionally, it should be noted that the optimized parameters of PBRTQC are not fixed and may vary across different laboratories. Furthermore, the introduction of a new testing system may necessitate the determination of new model parameters, thereby further complicating the maintenance of the PBRTQC system (*25*).

In conclusion, the AI-PBRTQC group was more efficient in identifying quality risks than the conventional PBRTQC. Artificial intelligence PBRTQC can also effectively identify quality risks in a small number of samples. AI-PBRTQC can be used in biochemistry and also in immunology. Quality risks such as reagent calibration, reagent machine time, and reagent brand replacement can be identified.

## Data Availability

The data used to support the findings of this study are available from the corresponding author upon request.
